# Impact of antifungal stewardship interventions on the susceptibility of colonized *Candida* species in pediatric patients with malignancy

**DOI:** 10.1038/s41598-021-93421-3

**Published:** 2021-07-08

**Authors:** Ali Amanati, Parisa Badiee, Hadis Jafarian, Fatemeh Ghasemi, Samane Nematolahi, Sezaneh Haghpanah, Seyedeh Sedigheh Hamzavi

**Affiliations:** 1 Professor Alborzi Clinical Microbiology Research Center, Shiraz University of Medical Sciences, Shiraz, Iran; 2 Head of Infection Control Unit, Amir Medical Oncology Hospital, Shiraz University of Medical Sciences, Shiraz, Iran; 3 Department of Biostatistics, Shiraz University of Medical Sciences, Shiraz, Iran; 4 The Hematology Research Center, Shiraz University of Medical Sciences, Shiraz, Iran

**Keywords:** Cancer, Microbiology

## Abstract

There is a worldwide concern regarding the antimicrobial resistance and the inappropriate use of antifungal agents, which had led to an ever-increasing antifungal resistance. This study aimed to identify the antifungal susceptibility of colonized *Candida* species isolated from pediatric patients with cancer and evaluate the clinical impact of antifungal stewardship (AFS) interventions on the antifungal susceptibility of colonized *Candida* species. *Candida* species colonization was evaluated among hospitalized children with cancer in a tertiary teaching hospital, Shiraz 2017–2018. Samples were collected from the mouth, nose, urine, and stool of the patients admitted to our center and cultured on sabouraud dextrose agar. The isolated yeasts identified by polymerase chain reaction–restriction fragment length polymorphisms (PCR–RFLP). DNA Extracted and PCR amplification was performed using the ITS1 and ITS4 primer pairs and Msp I enzyme. The broth microdilution method was used to determine the minimum inhibitory concentrations (MICs) for amphotericin B, caspofungin, and azoles. The prevalence of *Candida albicans* in the present study was significantly higher than other *Candida* species. *Candida albicans* species were completely susceptible to the azoles. The susceptibility rate of *C. albicans* to amphotericin B and caspofungin was 93.1% and 97.1%, respectively. The fluconazole MIC values of *Candida albicans* decreased significantly during the post-AFS period (*P* < 0.001; mean difference: 72.3; 95% CI of the difference: 47.36–98.62). We found that ‏52.5% (53/117) of the isolated *C. albicans* were azole-resistant before AFS implementation, while only 1.5% (2/102) of the isolates were resistant after implementation of the AFS program (*P* < 0.001). *C. albicans* fluconazole and caspofungin resistant rate also decreased significantly (*P* < 0.001) after implementation of the AFS program [26 (32.9%) versus 0 (0.0%) and 11 (10.9%) versus 1 (0.9%), respectively]. Besides, fluconazole use (*p* < 0.05) and fluconazole expenditure reduced significantly (about one thousand US$ per year) after the AFS program. Our results confirm the positive effect of optimized antifungal usage and bedside intervention on the susceptibility of *Candida* species after the implementation of the AFS program. *C. albicans* and *C. glabrata* exhibited a significant increase in susceptibility after the execution of the AFS program.

## Introduction

The prevalence of candidemia/invasive candidiasis (IC) is on the rise due to excessive usage of broad-spectrum antibiotics, indwelling catheters, HIV infection, malignancies, transplants, invasive procedures, and prolonged hospitalization, especially in intensive care patients and neonates^1–3^. More than 30 *Candida* spp. are recognized that they could infect humans^4^. Overall, 90% of IC are related to *C. albicans*, *C. glabrata*, *C. parapsilosis*, *C. tropicalis*, and *C. krusei*^5,6^.

Antifungal resistance usually occurs following selective pressure induced by the use or misuse of antifungal agents in high-risk patients, especially those with malignancy^7–9^. The epidemiology of IC could be affected by the type and duration of previous antifungal exposure, such as prolonged antifungal prophylaxis^10^.

Currently, the urgent need for an AFS program is well recognized and encouraged by many experts^11,12^. By optimizing antifungal use, including improving the selection and duration of antifungal therapy, potential economic saving also could be achieved^12,13^. These efforts objectively have been evaluated by different instruments such as total antifungal prescriptions, which defined by daily doses (DDDs) and days of therapy (DOTs)^12^; however, long term effects of AFS interventions such as potential effects on the epidemiology and the antifungal susceptibility patterns are less known. Although *C. albicans* is the most common cause of IC, the prevalence of non*-albicans* species increases^7^. The emergence of non*-albicans Candida* infections has become a global concern; however, as we described previously, change in the epidemiologic pattern could be possible after sustained adherence to the AFS program^14^. Similar positive effects could be expected on the susceptibility pattern of *Candida* species after AFS implementation. Therefore, this study aimed to identify the antifungal susceptibility of colonized *Candida* species isolated from pediatric patients with malignancy and investigate the ASP intervention effects on the antifungal susceptibility patterns.

## Methods

### Study design

This is a cross-sectional study investigating the susceptibility pattern of colonized *Candida* species in children with malignancy. Samples were collected from oral/nasal secretions and urine/stool specimens. Every eligible patient undergoes regular weekly sampling after admission until discharge. We used the original data from our previous study in Amir medical oncology center (AMOC), which was conducted before the implementation of AFS during 2011–2012 in colonized pediatric patients with malignancy (period-1; p1)^15^ to compare the clinical impact of AFS interventions on the antifungal susceptibility of colonized *Candida* species with our present study (period-2; p2). So, it should be mentioned that this study was designed to investigate the susceptibility of colonized *Candida* species before and after the implementation of AFS in a referral tertiary oncology center.

### Participants

In this study, children aged < 18-year-old with hematologic malignancy or solid organ tumors were included between 2017 and 2018. In children with severe thrombocytopenia or bleeding tendency, only urine and stool samples were collected.

### Mycological study

Samples were cultured on Sabouraud Dextrose Agar (Merck, Germany) medium and transferred to the mycology laboratory of Professor Alborzi Clinical Microbiology Research Center for identification and susceptibility testing. The isolated yeasts identified by polymerase chain reaction–restriction fragment length polymorphisms (PCR–RFLP)^16^. DNA Extracted and PCR amplification was performed using the ITS1 and ITS4 primer pairs (MWG-Biotech AG, Germany) and Msp I enzyme^17^. The isolated fungi were cultured twice on Potato Dextrose Agar (OXOID LTD, Basingstoke, Hampshire, England) medium at 35 °C for 24–48 h to ensure the purity of the isolates. *C. parapsilosis* ATCC-22019 and *C. krusei*-ATCC-6258 ‏were used as standard quality control CLSI-recommended strains.

### Antifungal susceptibility testing

The susceptibility testing of amphotericin B (AMB) and posaconazole (POS) (Sigma-Aldrich, Germany), caspofungin (CAS) fluconazole (FLU), itraconazole (ITR) and voriconazole (VOR) (Sigma-Aldrich, USA) were performed according to CLSI M27-A3^18^ and CLSI M27-S4^19^.

Briefly, RPMI 1640 medium (Sigma-Aldrich, England) with l-glutamine and 2% glucose was prepared. PH adjusted to 7.0. Inoculum’s suspension of each yeast (0.5 McFarland) was prepared using the spectrophotometric method at 530 nm. Serial dilution with RPMI was prepared for fluconazole from 0.125 to 64 μg/mL and other antifungal agents from 0.032 to 16 μg/m. Positive and negative control (wells without antifungals and wells without yeast) were considered for evaluating the tests. The MIC was read visually after 24 and 48 h. The MIC for POS, CAS, FLU, ITR, and VOR were described as the lowest concentration of antifungal agent could decrease fungal growth by 50% compared to positive controls. For AMB, complete growth inhibition was considered as MIC value. The wild-type species is a sensitive species that presents no mutation or acquires antifungal resistant gen. In resistant species (non-wild type), there is some resistant gen that exhibits a high MIC value. Epidemiological cut-off value (ECV) is defined as the MIC value at least 95% of wild-type isolates under this MIC value^20,21^. The MIC50, MIC90, and ECV of the isolated species and wild and non-wild species were calculated.

### Antifungal Stewardship program in Amir medical oncology center

AFS is a “strategic planning” that can be summarized in learning, training, and continuous practice to improve evidence-based skills in managing invasive fungal diseases (IFDs), including IC in high-risk patients. By the sustained adherence to the AFS, indiscriminate use of antifungal agents, drug resistance, side effects, and costs will be reduced. The AFS has been executed in our center since June 2015. Characteristics of AFS interventions are summarized in Table 1. It should be noted that the diagnosis and treatment of the IFDs were significantly improved after the implementation of the AFS. Changing from empiric therapy to pre-emptive antifungal treatment strategies was accomplished by the application of non-culture-based methods, such as galactomannan (GM) antigen, mannan, and polymerase chain reaction (PCR). Therapeutic drug monitoring and antifungal susceptibility testing have become the standard of care for monitor serum voriconazole concentrations and targeted therapy since early 2016.

**Table 1:** Main components of AFS interventions for the management of invasive fungal diseases (including invasive candidiasis and invasive aspergillosis) in Amir medical oncology center.

**Appropriate treatment of the suspected IFDs**
Disposition to targeted therapy (by diagnostic driven approach) instead of empiric treatment
Adherence to current evidence-based guidelines in the treatment of the IFDs instead of individual decision making
**Appropriate antifungal prescription**
Appropriate antifungal selection
Appropriate duration
Appropriate administration route
Appropriate dosage
Limited use of azoles for prophylaxis of the IFDs (only for secondary prophylaxis in patients with a previous history of IFDs)
Regular epidemiologic surveillance to estimate of fungal infection incidence and detection of any epidemiologic shift
Regular surveillance of the susceptibility pattern to antifungal drugs
Appropriate use of new diagnostic modalities (implementation of routine GM test, twice/week during prolonged and profound neutropenic phase (ANC < 500 cells/mm^3^)
Improving mycological diagnostic approach with judicious use of bronchoalveolar lavage and ultrasound/CT scan guided lung biopsy (or other organs as needed)
Time-sensitive automatic stop orders for specified antifungal prescriptions
Switching from intravenous to oral antifungal, when appropriate and confirmed by the infectious disease consultant
Full-time laboratory services (24-h, 7 days per week coverage) and strategies for reducing lab turnaround time (establishing a “hotline” for contributors to call about the lab test results)
**Non-medical approach to prevent fungal infections**
Applying modalities to reduce the nosocomial infections (for example, diminished colonization by the appropriate use of an indwelling catheter)
Surveillance of the possible environmental roots of infection (for example, surveillance of indoor spore load in the hospital’s wards)

*AFS* antifungal Stewardship, *IFDs* invasive fungal diseases, *IMDs* invasive mold diseases, *GM* galactomannan, *ANC* absolute neutrophil count, *AMOC* Amir Medical Oncology Center.

As we know, if the colonized fungi population contains some resistance strains, they will show resistance if exposed to antifungal drugs. In heterogenous fungi population after exposure to antifungal medications, resistance could be acquired by selection pressure^7^. Amphotericin will be the main culprit for antifungal prophylaxis, while fluconazole use has dropped dramatically during the second study period. Non-azole antifungal prophylaxis was implemented in our center to save last-line azole agents (voriconazole and posaconazole) for treating invasive mold infections. We test that our prophylaxis strategy could affect amphotericin resistance rate during the second study period.

### Statistical analysis

Data were analyzed using IBM SPSS Statistics 21 software (IBM Corp. Released 2012. IBM SPSS Statistics for Windows, Version 21.0. Armonk, NY: IBM Corp.). All categorical variables reported in percentages and numbers. P values calculated using the Chi-square test and Fisher’s exact test. P values < 0.05 considered being statistically significant. The Pearson correlation test was used to investigate the correlations between quantitative variables.

### Ethics and consent to participate

The study was approved by the medical ethics committee of *Professor Alborzi clinical microbiology research center*, Shiraz University of medical sciences, Iran (ID number: 94-01-49-11275). The authors confirm that all methods performed in accordance with the relevant guidelines and regulations. All individuals (or their parents) in the study population were informed about the current study, with written consents obtained before enrolment in the present study.

## Results

The incidence of IFDs was ranged from 7.7 to 12.5/1000 admissions during 2015–2018 in our center. Invasive candidiasis (IC) is the most common form of IFDs (47.2%), and its annual incidence range is 22.5–55.3%.

From May 2017 to November 2018, 482 specimens were collected from 136 pediatric patients with hematological malignancies or solid organ tumors. Most patients were male (53.3%), and the mean age was 7.57 years (Median: 6.5, Std. Deviation: ± 4.85, range from 4.8 months to 18 years). During this period, 36% of the studied cases were monitored for at least 4 weeks by weekly sampling, whereas 64% followed for more than four weeks.

Acute lymphoblastic leukemia (41/136, 30.1%), acute myeloblastic leukemia (18/136, 13.2%), and neuroblastoma (13/136, 9.5%) were the most common underlying diseases, respectively. In total, 51.4% (70) were neutropenic (absolute neutrophil count < 1500 cells/mm^3^).

Eighty-two cases were colonized with at least one *Candida* spp. and 133 strains of *Candida* species identified (two species not identified). The most prevalent isolated species was *C. albicans* (102 strains) followed by *C. krusei* (7), *C. kefyr* (7), *C. parapsilosis* (5), *C. glabrata* (4), *C. tropicalis* (3), and *C. famata* (3). The susceptibility of *Candida* species to different antifungal drugs summarized in Table 2.

**Table 2:** Susceptibility of 131 *Candida* spp. to antifungal drugs and distributions of MIC (µg/ml) by CLSI broth microdilution method.

Organism	AF	Breakpoints	S	SDD	I	R	ECV^a^	WT	N-WT	MIC50^a^	MIC90^a^	MIC range^a^
*C. albicans*	AmpB	S ≤ 1, R ≥ 1	93.1%	–	–	6.9%	4	96%	4%	0.250	0. 50	0.032–8
	CSF	S ≤ 0.25, I = 0.5, R ≥ 1	97.1%	–	1.96%	1%	0.25	97%	3%	0.032	0.064	0.032–1
	VCZ	S ≤ 0.12, I = 0.25, − 0.5 R ≥ 1	100%	–	–	–	0.032	98%	2%	0.032	0.032	0.032–0.125
	FCZ	S ≤ 2, SDD = 4, R ≥ 8	100%	–	–	–	0.25	98%	2%	0.032	0.125	0.032–4
	ITC	S ≤ 0.12, SDD = 0.25, − 0.5 R ≥ 1	100%	–	–	–	0.064	98%	2%	0.032	0.032	0.032–0.064
*C. glabrata*	AmpB	S ≤ 1, R ≥ 1	100%	–	–	–	0.25	75%	25%	0.250	0.5	0.25–0.5
	CSF	S ≤ 0.12, I = 0.25, R ≥ 0.5	75%	–	25%	–	0.125	75%	25%	0.125	0.25	0.064–0.25
	VCZ	ECV = 0.5, WT: MIC ≤ ECV & non-WT: MIC > ECV					0.032	100%	–	0.032	0.032	0.032
	FCZ	SDD ≤ 32, R ≥ 64	–	100%	–	–	0.25	75%	25%	0.25	1	0.125–1
	ITC	S ≤ 0.12, SDD = 0.25, − 0.5 R ≥ 1	100%	–	–	–	0.064	75%	25%	0.064	0.125	0.064–0.125
*C. krusei*	AmpB	S ≤ 1, R ≥ 1	100%	–	–	–	0.5	85.7%	28.6%	0.5	1	0.25–1
	CSF	S ≤ 0.25, I = 0.5, R ≥ 1	14.3%	–	57.1%	28.6%	0.5	71.4%	14.3%	0.5	1	0.25–1
	VCZ	S ≤ 0.5, I = 1, R ≥ 2	100%	–	–	–	0.125	85.7%	28.6%	0.125	0.25	0.064–0.25
	FCZ	*C. krusei* is considered resistant to FCZ, irrespective of the MIC					–	–	–	–	–	–
	ITC	S ≤ 0.12, SDD = 0.25, − 0.5 R ≥ 1	85.7%	14.3%	–	–	0.125	85.7%	28.6%	0.125	0.25	0.125–0.25
*C. tropicalis*	AmpB	S ≤ 1, R ≥ 1	100%	–	–	–	0.25	66.7%	33.3%	0.25	0.5	0.25–0.5
	CSF	S ≤ 2	100%	–	–	–	0.064	66.7%	33.3%	0.064	1	0.032–1
	VCZ	S ≤ 0.12, I = 0.25, − 0.5 R ≥ 1	100%	–	–	–	0.032	100%	–	0.032	0.032	0.032
	FCZ	S ≤ 8, R ≥ 64	100%	–	–	–	0.032	66.7%	33.3%	0.032	0.125	0.032–0.25
	ITC	S ≤ 0.12, SDD = 0.25, − 0.5 R ≥ 1	100%	–	–	–	0.032	100%	–	0.032	0.032	0.032
*C. parapsilosis*	AmpB	S ≤ 1, R ≥ 1	100%	–	–	–	0.25	80%	20%	0.25	0.5	0.032–0.5
	CSF	S ≤ 2, I = 4, R ≥ 8	100%	–	–	–	0.5	80%	20%	0.064	0.125	0.032–0.125
	VCZ	S ≤ 0.12, I = 0.25, − 0.5 R ≥ 1	80%	–	20%	–	0.032	80%	20%	0.032	0.5	0.032–0.5
	FCZ	S ≤ 2, SDD = 4, R ≥ 8	80%	–	–	20%	0.064	80%	20%	0.064	16	0.032–16
	ITC	S ≤ 0.12, SDD = 0.25, − 0.5 R ≥ 1	80%	20%	–	–	0.032	80%	20%	0.032	0.25	0.032–0.25

Based on recommended CLSI 24-h minimum inhibitory concentration limits.

*AmpB* Amphotericin B, *CSF* Caspofungin, *VCZ* Voriconazole, *FCZ* Fluconazole, *ITC* Itraconazole, *AF* antifungal, *SDD* susceptible dose-dependent, *S* sensitive, *I* intermediate, *R* resistant, *ECV* Epidemiological Cutoff Value; ECVs capture ≥ 97.5% of the statistically modelled population, *WT* Wild-type, *NWT* non-wild-type, *MIC50* Minimum Inhibitory Concentration required to inhibit the growth of 50% of organisms, *MIC90* Minimum Inhibitory Concentration required to inhibit the growth of 90% of fungal species.

^a^(µg/ml).

All *C. albicans* were susceptible to the azole antifungal agents. The susceptibility rate of *C. albicans* to amphotericin B and caspofungin was 93.1% (95) and 97.1% (99), respectively. All the *C. krusei* isolates were sensitive to amphotericin B and voriconazole; while, 28.6% were resistant to caspofungin. For itraconazole, 85.7% were sensitive, and 14.3% were susceptible dose-dependent. *C. parapsilosis* isolates were sensitive to amphotericin B, caspofungin. For itraconazole, 80% were sensitive, and 20% were susceptible dose-dependent. 80% of *C. parapsilosis* found to be susceptible to fluconazole. All *C. glabrata* and *C. tropicalis* isolates were sensitive to the tested antifungal agents.

The ECV, MIC50, and MIC90 for in vitro susceptibility testing of *Candida* spp. calculated (Table 2). Susceptibility of different antifungals to *C. kefyr* and *C. famata* is provided in Table 3. CLSI breakpoints are not available for *C. Kefyr* and *C. famata*.

**Table 3:** Susceptibilities of different antifungals to *C. kefyr* and *C. famata.*

Species (no. tested)	Antifungal agent	MIC (μg/ml)		
		Range	50%	90%
*C. kefyr* (7)	Fluconazole	0.032–0.25	0.064	0.125
	Voriconazole	0.032	0.032	0.032
	Itraconazole	0.032	0.032	0.032
	Caspofungin	0.032–1	0.064	0.125
	Amphotericin B	0.064–2	0.25	0.5
*C. famata* (3)	Fluconazole	0.032–0.25	0.032	0.032
	Voriconazole	0.032	0.032	0.032
	Itraconazole	0.032–0.25	0.032	0.032
	Caspofungin	0.032–0.125	0.032	0.032
	Amphotericin B	0.25–0.5	0.25	0.25

**Figure 1:**
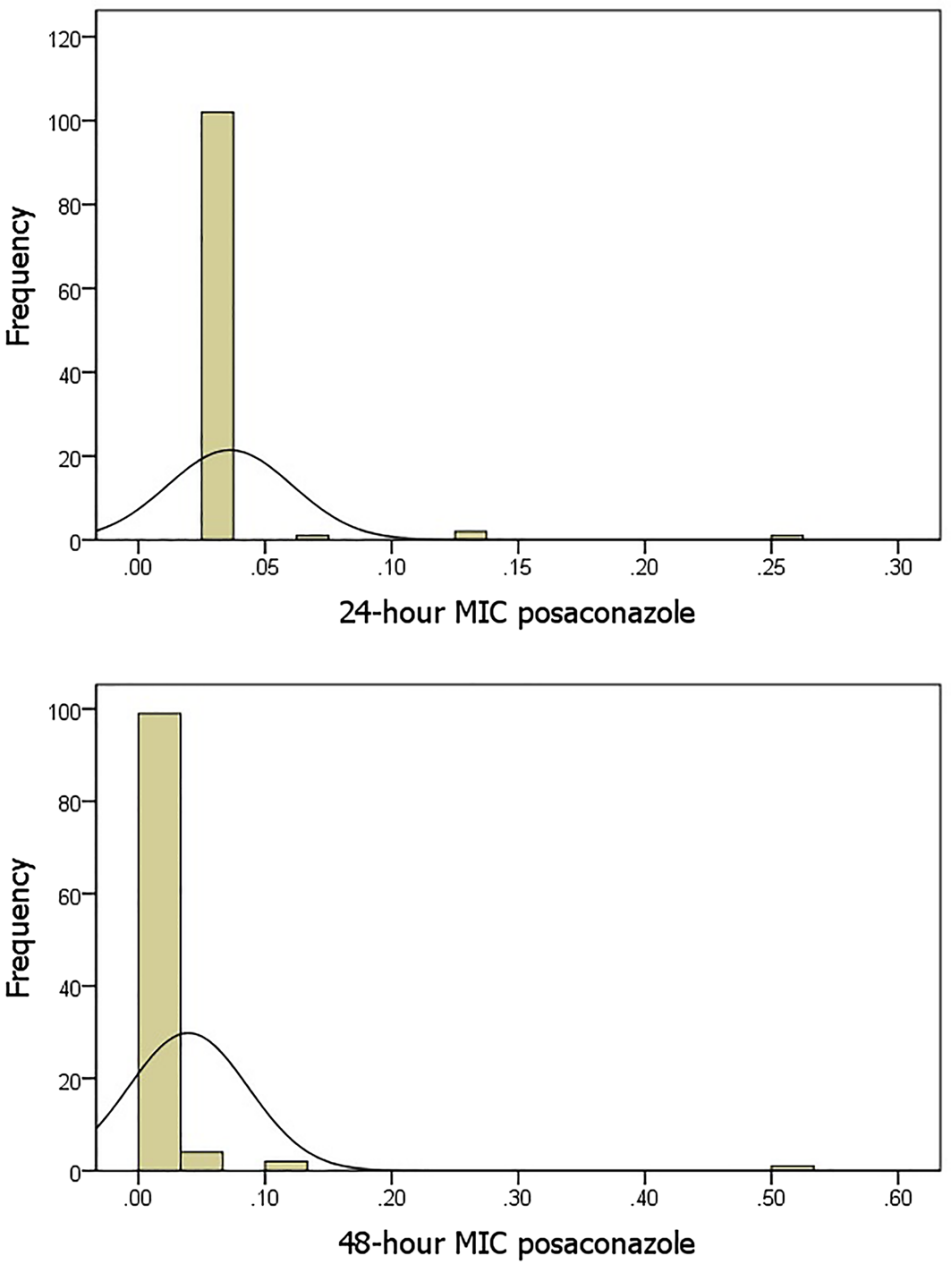
24-h and 48-h MIC distribution with a histogram of the isolated *C. albicans*.

CLSI does not provide posaconazole minimal inhibitory concentration breakpoint for *C. albicans*. Posaconazole 24-h and 48-h MIC statistics were determined for 102 *C. albicans* isolates. Accordingly, mean 24-h and 48-h MIC were 0.0361 and 0.0394, respectively (Table 4, Fig. 1).

**Table 4:** Posaconazole 24-h and 48-h MIC statistics for 102 isolates of *C. albicans.*

	24-h MIC	48-h MIC
Mean	0.0361	0.0394
Median	0.0320	0.0320
Mode	0.03	0.03
Std. deviation	0.02470	0.04727
Variance	0.001	0.002
Range	0.22	0.47
Minimum	0.03	0.03
Maximum	0.25	0.50

Epidemiological changes in the *Candida* colonization pattern described in our previous report. During period 1 (p1), 46.5% (88) of the studied cases (n = 188) were colonized, while in the 2^nd^ period, the colonization rate reached 59.9% (P value = 0. 0.017)^14^. In total, 25.3% (23) of the cases were receiving inpatient-antifungal prophylaxis during the 2nd period, mainly with the liposomal formulation of amphotericin B, while 54% were on antifungal prophylaxis during p1, mostly with fluconazole or itraconazole [Difference 21.2%, 95% CI 9.16–31.77%, P = 0.0007]. Despite a significant increase in the colonization rate, we found a significant reduction in non-*albicans* species colonization after the implementation of AFS. This success was achieved by controlling and restricting antifungal usage during p2.

In a study by Hadadi et al., which was conducted during 2011–2012 (p1) in our center, *C. albicans* was the most common species, followed by *C. krusei*, *C. glabrata*, *C. tropicalis*, *C. famata*, *C. parapsilosis*, *C. dubliniensis*, and *C. kefyr*. During p1, *C. glabrata* was the most resistant isolated *Candida* species, showing 70% resistant to fluconazole and 50% to itraconazole, 7.5% to amphotericin B, and 14% to ketoconazole^15^.

**Figure 2:**
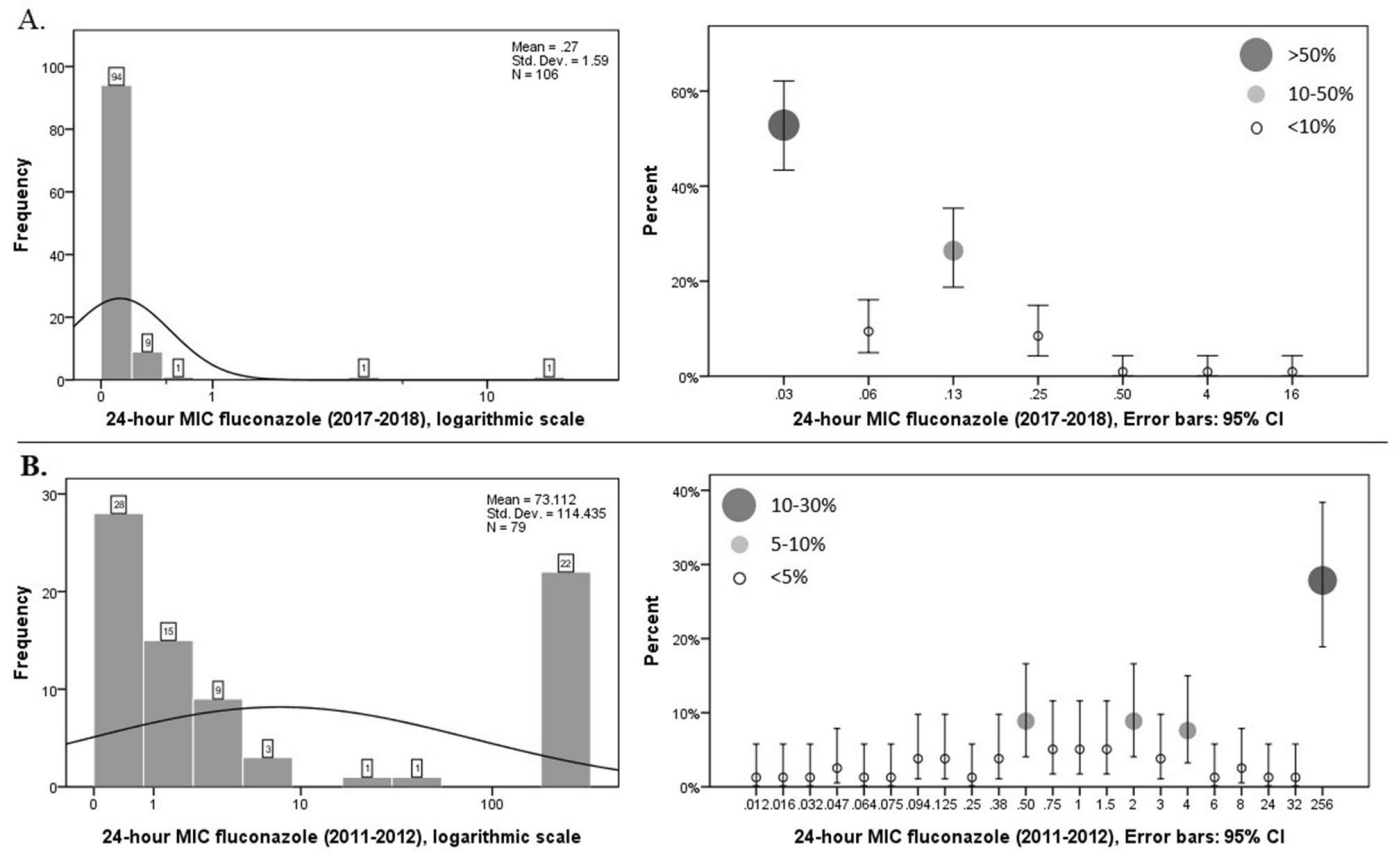
24-h MIC fluconazole of 117 (2011–2012), and 106 (2017–2018) strains of *C. albicans*. (**A**) Illustrate chart bar (left) which each bar is labeled with the number of isolates and logarithmic scales (right) of 24-h MIC fluconazole during p2 (2017–2018) which Frequency of MIC results is presented in error bars with 95% CI. Each error bar is labeled by circles that are representative of MIC frequency. (**B**) Illustrate chart bar (left) and logarithmic scales (right) of 24-h MIC fluconazole during p1 (2011–2012). MIC distribution histogram also is provided for better comparison between the two periods.

During p1, ‏52.5% (53/117) of the isolated *C. albicans* were found azole-resistant, while only 1.5% (2/102) of the isolates were azole-resistant during p2 (P value < 0.001). Amongst the 117 tested isolates of *C. albicans*, 52.5% (53) of the isolates were found to be azole-resistant during p1, while only 1.5% (2) were resistant during p2 (P value < 0.001). No fluconazole-resistant (MIC ≥ 8 μg/ml) *C. albicans* was detected during p2 (P value < 0.001). Multidrug-resistant strains, including azole, caspofungin, and amphotericin B resistant isolates, were not found within the two study periods (Fig. 2).

**Figure 3:**
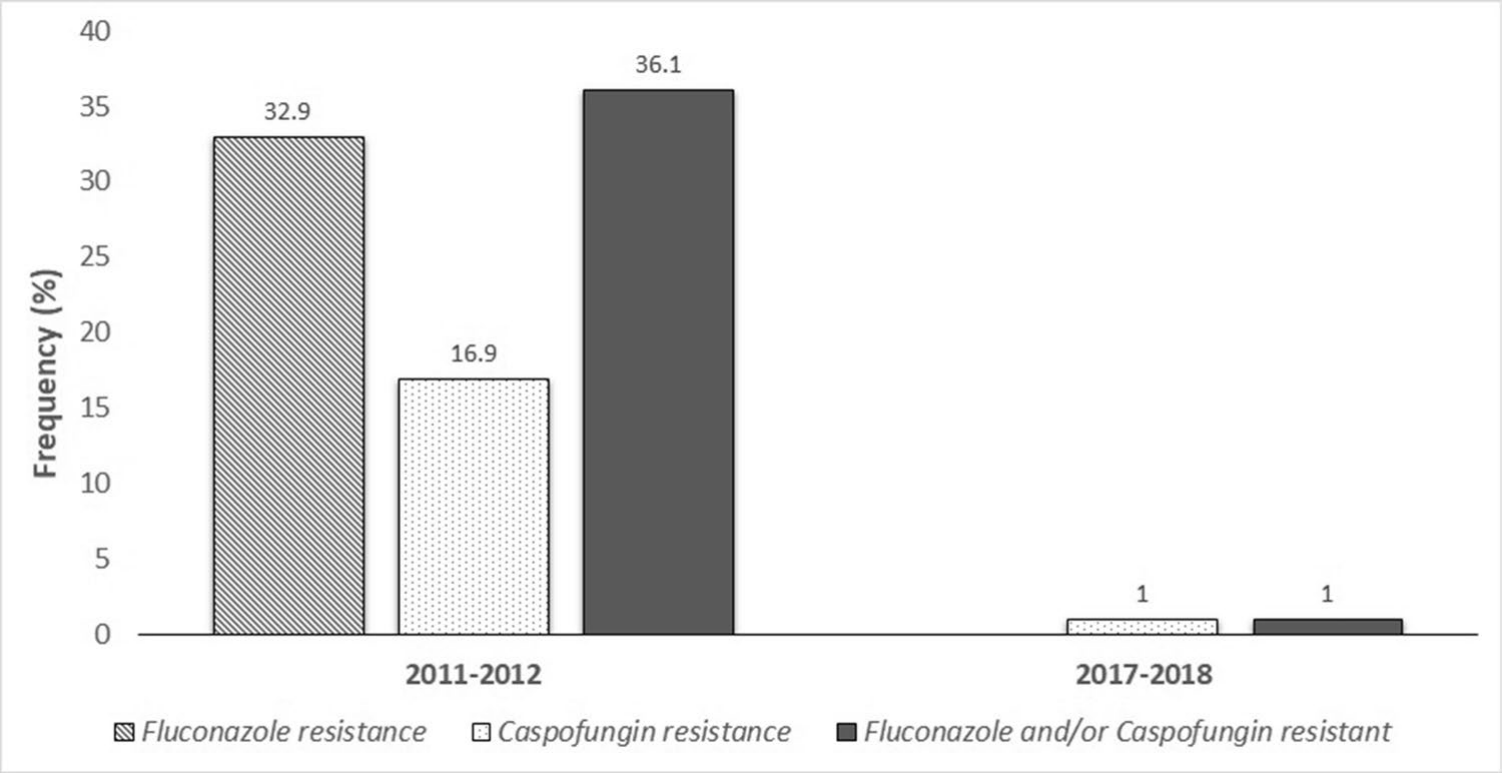
Frequency of fluconazole-resistant, caspofungin-resistant and fluconazole and/or caspofungin-resistant strains of *C. albicans* during the two study periods.

Despite the significant reduction in fluconazole and caspofungin-resistant, during p2, a slight increase in the incidence of amphotericin B-resistant *C. albicans* was detected (Table 5). This change could be explained by the antifungal preventive strategy shifting to liposomal amphotericin B since 2015. However, the frequency of amphotericin B-resistant *C. albicans* was not affected by liposomal amphotericin B prophylaxis between the two periods (*p* = 0.619) (Fig. 3).

**Table 5:** The susceptibility of isolated *C. albicans* against fluconazole, caspofungin, and amphotericin B, during 2011–12 (period 1) and 2017–2018 (period 2).

Antifungal agent	Susceptibility	Period 1	Period 2^a^	p-value
Fluconazole	Sensitive	53 (67.1)	102 (100)	< 0.001**
	Resistant	26 (32.9)	0	
Caspofungin	Sensitive	94 (89.5)	101 (99.1)	< 0.001**
	Resistant	11 (10.9)	1 (0.9)	
Amphotericin B	Sensitive	83 (100)	95 (93.1)	< 0.001**
	Resistant	0	7 (6.9)	

^a^Number (%) of children colonized with *C. albicans.*

*No fluconazole-resistant isolates of *C. albicans* was found during period 2 (2017–2018).

**Statistically significant by Fisher’s exact test.

**Figure 4:**
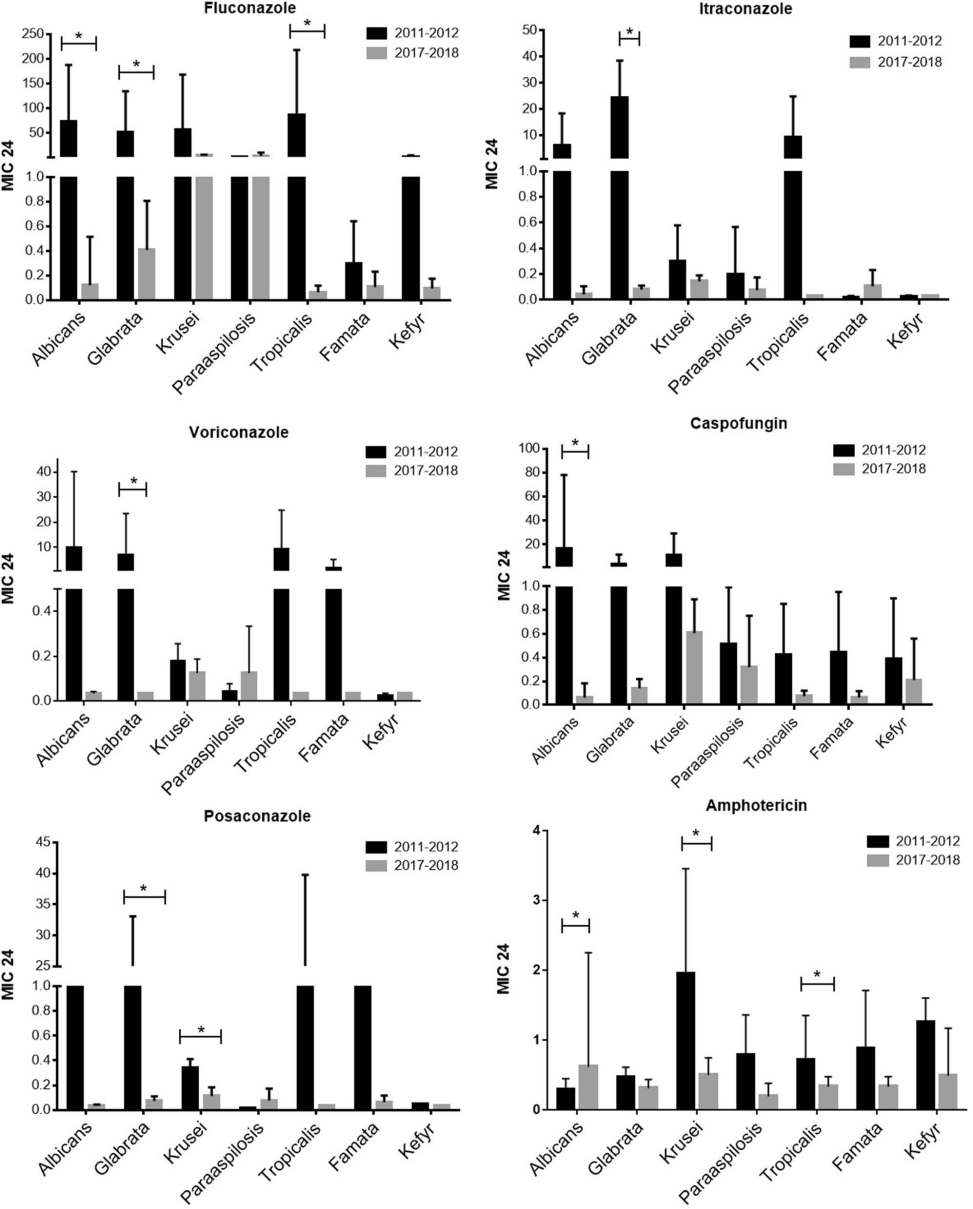
The mean MIC value (24-h) of *C. albicans*, *C. glabrata*, *C. krusei*, *C. parapsilosis*, *C. tropicalis*, *C. famata*, and *C. kefyr* for fluconazole, itraconazole, voriconazole, caspofungin, posaconazole, and amphotericin B, during the two study periods. Error bars represent standard deviations. *P ≤ 0.05 by the two-way ANOVA test.

We also analyzed the rate of fluconazole, voriconazole, itraconazole, caspofungin, and amphotericin B resistance amongst the non*-albicans* colonized species between the two study periods. A significant decrease in fluconazole, itraconazole, and caspofungin resistance was found among the *C. glaberata* strains during the second study period (p2) compared with 2011–2012. Also, a statistically significant reduction in amphotericin B resistance (*p* = 0.007) found during p2 in *C. krusei* isolates (Fig. 4).

We also review our available data for fluconazole usage (including multiple courses of fluconazole prescriptions per patient) before and after implementing the AFS program and their impact on health economics. During the last year before the initiation of the AFS program (2014–2015), fluconazole prescribed for 161 patients (total admissions: 4975), while during the first year of the AFS program (2015–2016), fluconazole administrated in 92 cancer patients (total admissions: 5706). The fluconazole consumption showed significant decrease (*p* < 0.001) from 3.2 to 1.6% (33 in 1000 cases to 16 in 1000 cases). No significant changes observed in the crude mortality rate after implementing the AFS program (0.7% versus 0.5%, respectively; *p* = 0.471). The total cost of the fluconazole usage was also reduced by 610 US$ after the start of the AFS program (6099 US% versus 5189 US%, *p* = 0.164). Notably, mean days of prescription for each patient who received fluconazole increased during p2 to 36/1000 patients (3.37 days; SD: ± 2.67) compare to the p1 16/1000 patients (2.58 days; SD: ± 3.035), which is statistically significant (*p* = 0.0315).

## Discussion

Amongst the 136 studied cases, 60% were colonized with at least one *Candida* species. Most of them were colonized with *C. albicans*, while *C. krusei*, *C. kefyr*, *C. glaberata*, *C. parapsilosis*, *C. tropicalis*, and *C. famata* were the least common *Candida* species. Our finding is in agreement with other reports on *Candida* colonization in children with malignancy^22–24^. Detailed information regarding the colonization pattern of the studied cases can be found in our recently published paper^14^.

Most *Candida* bloodstream infections, including central line-associated candidemia, originate from endogenous host flora^25–27^. The clinical impact of *Candida* colonization on the short-term mortality rate of patients with hematological malignancies has been documented in previous reports^28,29^. Higher mortality rates have been detected in patients with non-albicans species, such as *C. glabrata*, *C. kefyr*, and *C. krusei*, compared with *C. albicans*^28,30^. As discussed earlier, during p1, more than 35% of cases were colonized with non-*albicans* species, mostly *C. glabrata* and *C. krusei*. However, after implementing the AFS, non-*albicans* colonization decreased to less than 20%, mostly *C. krusei* and *C. Parapsilosis*, with a significant decrease in *C. glabrata* colonization^14^. *C. glabrata* is considered the second most common gastrointestinal yeast flora after *C. albicans*^31^. While an epidemiological shift from *C. albicans* to non-*albicans* species has been observed mainly in patients with hematological malignancies^32^, our recent survey confirmed that the successful implementation of AFS programs could reverse this shift.

We found full azole susceptibility of the isolated *C. albicans* in addition to 99% and 93% susceptibility to caspofungin and amphotericin B, respectively. Compare to other reports from our region; this study showed better susceptibility of colonized *C. albicans* to fluconazole and caspofungin^9,24^. Our finding confirmed that the AFS program (including amphotericin prophylactic strategy) could save azole antifungals as a first-line choice for IC.

In the present study, all clinical isolates of *C. krusei*, *C. glaberata*, *C. parapsilosis*, and *C. tropicalis* isolates were susceptible to amphotericin B (the most active agent for the treatment of non-albicans *Candida* species). Notably, despite our changing prophylactic strategy, much better susceptibility to amphotericin B was detected for isolated *C. krusei* as the most common non-albicans *Candida* species. Similar studies in our country shown 38.5–40% resistance to isolated *C. krusei* in colonized patients^9,24^. We found a higher resistance rate against caspofungin in isolated *C. krusei* (28.6%) compare to the previous reports in different parts of Iran^9,15,24,33^. The emergence of echinocandin-resistant *C. krusei* may be a paramount concern given the high MIC to fluconazole and voriconazole^32^.

Accordingly, amphotericin B can be considered the most active agent for treating non-albicans *Candida* species, especially *C. krusei* and *C. glaberata* in our study. Also, in this study, the colonized isolates of *C. Kefyr* and *C. famata* were susceptible to the tested antifungal agents.

In addition to the susceptibility results, we also compared the mean MIC value of each antifungal drug for *C. albicans*, *C. glabrata*, *C. krusei*, *C. parapsilosis*, *C. tropicalis*, *C. famata*, and *C. kefyr* during two study periods (Fig. 4). As shown in Fig. 4, a significant reduction in mean FCZ-MIC found for *C. albicans*, *C. glabrata*, and *C. tropicalis* in p2 compare to p1.

Based on our obtained results, all *C. albicans* isolates were susceptible to the tested azoles. Besides, these clinical isolates showed high susceptibility to amphotericin B and caspofungin (93.1% and 97.1%, respectively). Compare to the previously reported *C. albicans* susceptibility, which performed on the various clinical samples collected during 2005–2010, in Shiraz; higher azole-susceptibility was found in this study for *C. albicans* isolates, while susceptibility to amphotericin B and caspofungin (93% and 98.2%, respectively)^6^ remained unchanged.

At a global level, in some regions such as South Africa (African Region) and Taiwan, China (Western Pacific Region), fluconazole resistance *C. albicans* more frequently reported^34^. Fluconazole resistance *C. albicans* could be considered a predictor of cross‐resistance between azoles, especially in those with prior exposure to this antifungal class^35,36^. Cross-resistant between azoles and echinocandins among *Candida* species is another concern^37,38^.

As we have shown in this report, stewardship program is an efficacious approach for optimizing the use of antifungal drugs and improving azole susceptibility against *Candida* species, which could be achieved successfully by judicious AFS guideline adherence and facility-specific treatment recommendation monitoring.

As we mentioned earlier in the results section, the frequency of amphotericin B-resistant *C. albicans* was not affected by liposomal amphotericin B prophylaxis between the two periods. Our finding is promising compared with other reports concerning the change in resistance patterns of *Candida* species from fluconazole resistance to echinocandins resistance and the emergence of multidrug-resistant *Candida* species by increased therapeutic use of echinocandins^39^. The emergence of azole-resistant *C. glabrata* is also a concern in the setting that uses fluconazole prophylaxis^10^. In addition, resistance to amphotericin B remains relatively uncommon among *Candida* species^40,41^.

IFD continues to make a substantial economic burden on the oncology centers^42^. Many reports confirmed the benefits of AFS programs on the IFD-attributable hospital costs and reducing toxicities of antifungal agents^13^. Although, the clinical impact of AFS on the susceptibility of invasive fungi has not been investigated thoroughly, especially in high-risk cancer patients^11,43^. Given the emergence of antifungal resistance *Candida* species, appropriate use of antifungals and implementation of AFS programs is of utmost importance.

Therapeutic options for fungal infections are limited even before the global rise of antifungal resistance^34,44^; hence, a judicious prescription of available choices, especially non-azole antifungals, should be considered in high-risk settings, such as oncology centers. As we summarized in Table 1, our AFS program contains different strategies for optimizing antifungal drug prescription in patients suspicious of invasive forms of candidiasis and aspergillosis. Some examples are mentioned here for a better explanation. Before the beginning of the AFS program, febrile neutropenic patients universally receiving empiric antifungal therapy after 3–5 days of sustained fever without judicious use of state-of-the-art available diagnostic tests, including non-invasive tests such as automated blood culture systems, specific none culture-based mycologic assays (such as fungal polymerase chain reaction, galactomannan, and mannan) and interventional diagnostic modalities such as bronchoscopy/bronchoalveolar lavage, CT/ultrasound-guided lung biopsy, and sinus/skin biopsy. Indeed, our approach to febrile neutropenia changed from an empiric approach to a diagnostic-driven approach or pre-emptive treatment as suggested by experts and newer guidelines^45–50^. As we mentioned earlier in the result section, fluconazole prescription decreased per patient/1000 admission/year during the post-AFS period, but with the correct dose and duration. It should be noted that the AFS interventions should not put the cancer patient at greater risk of IFD, and a wise prescription of AF agents (formulary restrictions) should be weighed against high case-fatality rates of IFDs.

Our antifungal prophylaxis strategy changed after June 2015 to the liposomal formulation of amphotericin B. Particularly, antifungal prophylaxis alone is not fully effective without using air filtration system through high-efficiency particulate air filtration (HEPA) filters^51^; however, due to limited financial resources for providing HEPA filters, on-going hospital construction, potential risk of azole-resistant fungi, limited available new-azoles (posaconazole, isavuconazole, and ravuconazole) and echinocandins (micafungin and anidulafungin), and also increased number of invasive mucormycosis^52^, AFS team decide to use liposomal amphotericin B for antifungal prophylaxis. It should be reemphasized that amphotericin is routinely not recommended as systemic antifungal prophylaxis^53^; however, it should be noticed that liposomal amphotericin is not included in studies comparing amphotericin versus fluconazole^53^ and, so, a liposomal formulation of amphotericin B may be used in high-risk pediatric patients recommended by guidelines^47,50^. Besides, azole prophylaxis has a critical role in developing either unsusceptible strains or selecting intrinsic azole-resistant yeasts, such as *C. krusei*^7,10,54,55^.

In addition to the strategy mentioned above, we found that fluconazole had overused for treatment of the fungal mucositis (as one of the most common infectious complications during or after chemotherapy) which successfully replaced with nystatin and amphotericin-B mouth wash in non-severe cases (WHO grade I and II) who tolerate gargling. Prevention of unnecessarily prolonged catheterization and implementing bundled strategies for preventing central line-associated bloodstream infections (CLABSI) are other examples for preventing IC in our center.

There are lots of data concerning the positive effect of stewardship programs on bacterial resistance^56–59^; however, antifungal resistance is more challenging to measure due to its multi-factorial development. Even in colonized patients, susceptibility patterns might change over time, especially in immunocompromised hosts^9^. Although the AFS program has known short-term effects (e.g., reduction in antifungal consumption, costs, and outcomes) on the management of IFDs and patient safety^13,43,60,61^, its long-term effects have been described on resistance patterns^62^. Based on stewardship program metrics, change in resistance patterns and pathogen-specific resistance is the most challenging target^56^. There are scarce reports on the improvement of antifungal susceptibility of *Candida* species overtimes after the implementation of the AFS program to the best of our knowledge. Hence, the results of our study highlight the importance of strict adherence to the stewardship programs amongst cancer patients.

The small number of samples limited this study. Additionally, further studies using next-generation sequencing are needed to detect AFS program effects on antifungal resistance genes in *Candida* species.

In conclusion, *C. albicans* are the most prevalent colonizer among pediatric patients with malignancy, and azoles remain the most effective choice when used wisely. Improving *Candida* species antifungal susceptibility after the implementation of AFS is promising. Knowledge of etiologic agents and the regular identification of antifungal susceptibility patterns are necessary for oncology settings.

## Data Availability

The datasets used and/or analyzed during the current study are available from the corresponding authors on reasonable request.
